# Limited Outbreak of Highly Pathogenic Influenza A(H5N1) in Herring Gull Colony, Canada, 2022

**DOI:** 10.3201/eid2910.230536

**Published:** 2023-10

**Authors:** Liam U. Taylor, Robert A. Ronconi, Hayley A. Spina, Megan E.B. Jones, C. Brandon Ogbunugafor, Andrea J. Ayala

**Affiliations:** Yale University, New Haven, Connecticut, USA (L.U. Taylor, C.B. Ogbunugafor, A.J. Ayala);; Canadian Wildlife Service, Dartmouth, Nova Scotia, Canada (R.A. Ronconi);; Environment and Climate Change Canada, Dartmouth (R.A. Ronconi);; University of Guelph, Guelph, Ontario, Canada (H.A. Spina);; University of Prince Edward Island, Charlottetown, Prince Edward Island, Canada (M.E.B. Jones);; Canadian Wildlife Health Cooperative, Charlottetown (M.E.B. Jones)

**Keywords:** highly pathogenic influenza A(H5N1) virus, *Larus* *argentatus* subsp. *smithsonianus*, American herring gulls, avian flu, avian influenza virus, bird flu, HPAI, H5N1, influenza in birds, interclass transmission, poultry diseases, gull, seabird, influenza, respiratory infections, viruses, zoonoses

## Abstract

In summer 2022, highly pathogenic influenza A(H5N1) virus reached the herring gull (*Larus argentatus* subspecies *smithsonianus*) breeding colony on Kent Island, New Brunswick, Canada. Real-time monitoring revealed a self-limiting outbreak with low mortality. Proactive seabird surveillance is crucial for monitoring such limited outbreaks, protecting seabirds, and tracing zoonotic transmission routes.

Highly pathogenic avian influenza (HPAI) viruses pose a near-term threat to commercial poultry and a long-term risk for human pandemics (*1*,*2*). Recent outbreaks of HPAI A(H5N1) virus have also caused mass mortality events in vulnerable seabird populations (3). Because outbreaks are difficult to predict, knowledge of HPAI in wild birds is often limited to cross-sectional surveillance or post hoc records of mass mortality events (*4*–*6*).

Beginning in December 2021, an HPAI H5N1 virus strain spread from Eurasia into Canada, subsequently infecting wild, commercial, and backyard bird populations across North America (*4*) (https://www.usgs.gov/centers/nwhc/science/distribution-highly-pathogenic-avian-influenza-north-america-20212022). During summer 2022, we studied the life history of American herring gulls (*Larus* *argentatus* subspecies *smithsonianus*) at the Kent Island breeding colony in New Brunswick, Canada (Figure 1). Thus, we had an unusual opportunity to monitor emerging disease symptoms and deaths in a wild population. We report timelines, clinical details, and epidemiologic observations from a laboratory-confirmed HPAI outbreak that caused a relatively low death rate within a seabird colony.

**Figure 1:**
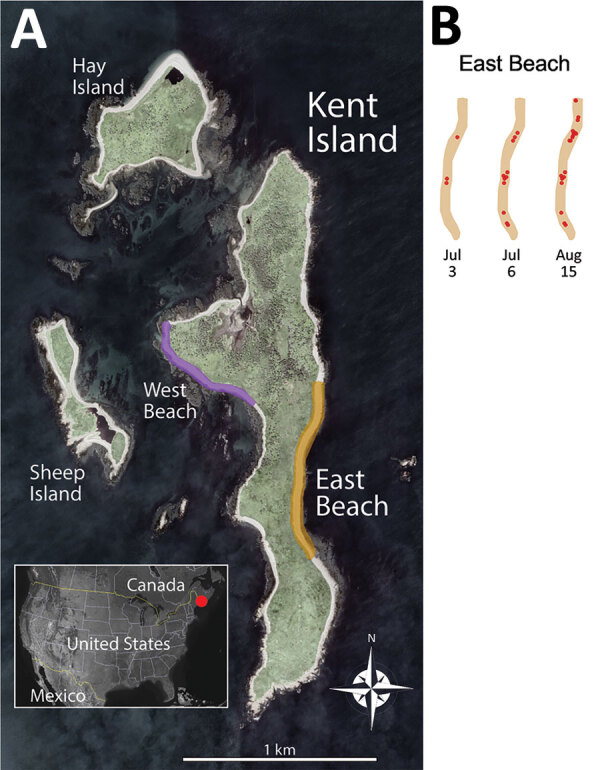
Location of gull breeding colony on Kent Island, New Brunswick, Canada, in study of limited outbreak of highly pathogenic influenza A(H5N1) in herring gull colony, 2022. A) Inset shows location of Kent Island in Canada. The main study site was on East Beach (yellow strip), and a secondary site was on West Beach (purple strip). Intermittent monitoring occurred across Kent, Hay, and Sheep Islands. Satellite image from Google Earth (https://earth.google.com). B) Accumulating carcass locations (red points) for 3 timepoints on East Beach.

## The Study

We monitored herring gulls on Kent Island (latitude 44.5828°N, longitude 66.7568°W; Figure 1). Gulls nest across the ≈100-ha island and on adjacent Hay and Sheep Islands (Figure 1). Herring gulls on Kent Island generally migrate north from eastern North America in early May, lay eggs in mid-May, hatch chicks in mid-June, and fledge chicks in August (*7*,*8*).

During June 1–August 15, 2022, we surveyed the main study area on East Beach (Figure 1) 1–3 times/day, conducting full census counts, monitoring disease symptoms, and individually marking carcasses. Other areas of Kent Island were surveyed on an intermittent schedule (Table 1). We assumed that all generally intact adult carcasses were virus-induced deaths because sudden deaths of adult birds are rare in breeding colonies. Because injuries and deaths are common among chicks, we were unable to assess virus-induced deaths in chicks except for suspected cases C1–C3 (Appendix).

**Table 1:** Summary of influenza-related herring gull deaths on Kent Island and neighboring islands in study of limited outbreak of highly pathogenic influenza A(H5N1), Canada, 2022*

Area	Census count† (+SD)	No. censuses conducted	No. deaths	Death rate,‡ %	Survey schedule
Kent Island, East Beach	526 (169)	110	22	4.2	1–3×/d, Jun 1–Aug 15, Sep 2
Kent Island, West Beach	221 (60)	88	15	6.8	1–2×/d, Jun 1–Jul 7, Jul 22–Aug 14, Sep 2
Kent Island, total§	3,077 (937)	10	66–87	2.1–2.8	1×/wk, Jun 1–Jul 7, Jul 15 (partial), Jul 22–Aug 14, Sep 2 (partial)
Sheep Island	900	1	25	2.8	Jul 5 (boat count), Jul 14 (partial), Aug 29
Hay Island	617 (267)	2	41	6.7	Jun 21, Aug 7

*Mean counts are of adult herring gulls >10 months of age. Partial surveys covered only part of the survey area. †Census counts varied with time, tide, and seasonal fluctuations for both breeding and nonbreeding populations. ‡Calculated according to mean census count. Our assumption that all intact adult gull carcasses were influenza-related might have raised death rate estimates, whereas intermittent surveys and imperfect carcass detection might have lowered death rate estimates. §Ranges for number of deaths and death rate are provided because of possible double counting of untagged carcasses by 2 observers outside of the main study sites.

**Figure 2:**
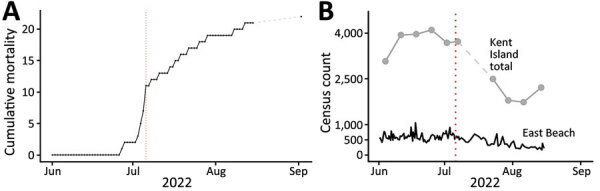
Adult herring gull deaths (cumulative, end-of-day) on East Beach, Kent Island, New Brunswick, Canada, in study of limited outbreak of highly pathogenic influenza A(H5N1) in herring gull colony, 2022. A) Cumulative mortality of herring gulls on East Beach during summer 2022. B) Census counts (number of breeding and nonbreeding adult herring gulls) from 1–3 surveys/day on East Beach and weekly total counts from surveys of the entire island. Red dotted lines mark July 6, the date of maximum gull deaths on East Beach.

We did not observe illness in the colony during a preliminary visit to Kent Island (May 24–27). On the morning of June 27, we spotted a lethargic adult herring gull on East Beach that died that afternoon (Table 2; Figure 2). Disease symptoms and deaths spiked at 9 deaths during July 4–8 (Figure 2). We observed 9 more deaths that accumulated more slowly through August 15; a final check on September 2 revealed only 1 new carcass. The total number of East Beach cases was 25, resulting in 22 confirmed deaths (4.2% site mortality; Tables 1, 2). Daily checks of West Beach for part of the summer showed a similar timeline and effect as that observed on East Beach (Tables 1, 2). Total carcass counts across Kent, Sheep, and Hay Islands indicated a <10% mortality rate (Table 2).

**Table 2:** Putative highly pathogenic influenza A(H5N1) virus cases in herring gulls on East Beach and West Beach study areas in study of limited outbreak on Kent Island, Canada, 2022*

Case no.	Location	Age,† y	First seen sick			Last seen alive			Found dead	
			Date	Time		Date	Time		Date	Time
1	East Beach	>4	Jun 27	≈09:30		Jun 27	≈09:30		Jun 27	≈16:30
2	East Beach	>4	NA	NA		NA	NA		Jun 28	≈17:00
6	East Beach	>4	Jul 2	12:11		Jul 2	12:11		Jul 3	09:38
8	East Beach	>4	Jul 3	09:49		Jul 5	13:22		Jul 5	17:52
11	East Beach	>4	Jul 3	20:30		Jul 3	20:30		Jul 4	08:57
12	East Beach	>4	Jul 4	09:14		Jul 4	14:10		Jul 5	08:46
15	East Beach	>4	Jul 4	13:51		Jul 4	17:09		Missing	NA
16	East Beach	>4	Jul 4	14:21		Jul 5	13:22		Jul 6	12:01
18	East Beach	4	NA	NA		NA	NA		Jul 4	17:13
20	East Beach	>4	Jul 5	08:51		Jul 6	16:46		Jul 6	19:39
24	East Beach	>4	Jul 5	17:45		Jul 5	18:18		Jul 6	11:34
26	East Beach	>4	Jul 5	17:10		Jul 6	11:36		Missing	NA
27	East Beach	4	Jul 6	11:54		Jul 6	19:53		Recovered?	NA
28	East Beach	>4	NA	NA		NA	NA		Jul 6	11:58
31	East Beach	>4	Jul 8	16:23		Jul 8	16:23		Jul 8	19:50
32	East Beach	>4	Jul 10	16:30		Jul 10	19:45		Jul 11	16:42
33	East Beach	>4	NA	NA		NA	NA		Jul 15	16:06
34	East Beach	>4	NA	NA		NA	NA		Jul 17	16:25
35	East Beach	>4	NA	NA		NA	NA		Jul 19	16:30
36	East Beach	>4	Jul 21	07:39		Jul 21	16:26		Jul 22	09:46
37	East Beach	>4	Jul 25	13:45		Jul 25	13:45		Jul 26	10:28
38‡	East Beach	>4	Jul 26	10:25		Jul 26	10:25		Jul 29	08:36
39	East Beach	1	NA	NA		NA	NA		Aug 8	10:13
40	East Beach	1	NA	NA		NA	NA		Aug 12	08:13
NA	East Beach	NA	NA	NA		NA	NA		Sep 2	NA
4	West Beach	4	NA	NA		NA	NA		Jul 1	11:45
5	West Beach	>4	Jul 1	16:18		Jul 1	16:18		Jul 1	17:20
9	West Beach	>4	Jul 3	16:41		Jul 3	16:41		Jul 4	10:20
10	West Beach	>4	Jul 3	16:43		Jul 3	16:43		Jul 4	10:25
13	West Beach	>4	Jul 4	10:04		Jul 4	10:04		Jul 4	14:49
14	West Beach	>4	NA	NA		NA	NA		Jul 4	10:11
19	West Beach	>4	NA	NA		NA	NA		Jul 5	09:46
22	West Beach	>4	NA	NA		NA	NA		Jul 5	17:15
25	West Beach	>4	NA	NA		NA	NA		Jul 5	16:20
NA	West Beach	>4	NA	NA		NA	NA		Jul 7	NA
NA	West Beach	>4	NA	NA		NA	NA		Jul 7	NA
NA	West Beach	>4	NA	NA		NA	NA		Jul 23	NA
NA	West Beach	NA	NA	NA		NA	NA		Sep 2	NA
NA	West Beach	NA	NA	NA		NA	NA		Sep 2	NA
NA	West Beach	NA	NA	NA		NA	NA		Sep 2	NA


*Case details are provided in the Appendix. Some carcasses were not numbered. NA, not applicable. †Estimated according to plumage (*8*) when noted. ‡Carcass was not discovered until what appeared to be several days after death.

During the summer 2022 breeding season, colony populations declined beginning in July (Figure 2). We assume that gulls exited the breeding colony because of normal seasonal phenology (*8*) rather than off-site deaths. Boat surveys of the surrounding Grand Manan archipelago (mid-June, mid-July) noted only 3 dead adult herring gulls in the water, and no mass mortality was reported on nearby beaches (1 dead HPAI virus–positive herring gull was found on Grand Manan on July 4; https://cfia-ncr.maps.arcgis.com/apps/dashboards/89c779e98cdf492c899df23e1c38fdbc). Censuses in June 2023 confirmed that the Kent Island herring gull population had returned for another breeding season (mean 4,290 herring gulls).

We collected case descriptions, images, and videos of herring gull adults and chicks from Kent Island (Appendix). Putative HPAI clinical signs in herring gulls matched those observed after experimental inoculations of HPAI H5N1 in related species (*9*,*10*). Neurologic symptoms progressed from lethargy and drooped wings to incoordination, head tremors, torticollis, and immobility over the course of hours or days. During the peak of the outbreak, dozens of additional birds displayed putative minor symptoms (e.g., slumped postures, hesitancy to fly) that were difficult to track and could not be linked to subsequent death. One bird manifesting severe neurologic distress apparently recovered within hours (case 27).

We collected 3 carcasses of adult symptomatic birds (case 8, case 20, and 1 euthanized bird in southwest Kent Island on July 15) along with 3 chicks (cases C1–C3). Carcasses were collected under Canadian Wildlife Service permit no. SS2506 (to R.A.R.). All 3 adults and 1 chick (case C2) tested positive for a Eurasian strain of HPAI H5N1 virus (Appendix).

All sick or dead adult gulls throughout June and July were >4 years old according to plumage, matching the usual minimum breeding age for the species (Table 2) (*8*). Plumage-based censuses suggested 3%–6% of colony birds were 1–3 years of age (data not shown). Those younger birds were not breeding, and only 2 were found dead on East Beach later in the summer (Table 2). From 16 fully-tracked cases (Table 2) and surveys conducted 1–3 times/day, we showed the mean time (+SD) from first seen sick to last seen alive was 7.8 +15.0 hours; the mean time from first seen sick to found dead was 20.9 +14.9 hours.

We calculated the basic reproduction number (R_0_) by using daily East Beach incidence data (June 1–August 15), gamma-distributed generation times from poultry data (4.8 +0.58 days) (*11*), and the exponential growth rate method from the R package R0 (*12*). Overall R_0_ was 1.02 (95% CI 0.95–1.11). R_0_ was 8.23 (95% CI 3.97–21.11) if estimated from the rising incidence period (June 1–July 6) but fell to 0.84 (95% CI 0.64–1.07) if estimated from the falling incidence period (July 7–August 15).

HPAI was suspected or confirmed in 4 other species breeding on Kent Island (Appendix): great black-backed gulls (*Larus marinus*), Canada geese (*Branta canadensis*), common eiders (*Somateria mollissima*), and American crows (*Corvus brachyrhynchos*). Unlike the mostly intact gull carcasses on Kent Island (Appendix Table 1), many carcasses on Hay Island were partially consumed. Likely predators or scavengers were great black-backed gulls and bald eagles (*Haliaeetus leucocephalus*). Beginning in July, we noted gray seals (*Halichoerus grypus*) loitering offshore at East Beach. Seals rarely interacted with adult seabirds but harassed herring gull chicks paddling from shore.

## Conclusions

A Eurasian lineage of HPAI H5N1 virus swept through the Kent Island herring gull colony starting in late June 2022. The outbreak appeared to slow within weeks (Figure 2) and resulted in <10% apparent colony mortality rate (Table 1). Low carcass disturbance (Appendix Table 1) and disease resistance or recovery (case 27) might have limited HPAI virus infections in the gulls. Furthermore, our islandwide censuses suggest 2022 population sizes were <25% of historical size across the same island area (Table 1) (*13*). Low densities might have reduced intraspecific transmission by limiting social interactions with infected conspecifics. However, we observed possible interspecific exposure routes through cohabitation (e.g., common eiders), predation/scavenging (e.g., bald eagles), and interactions between chicks and marine mammals (e.g., gray seals). Those pathways are consistent with global HPAI virus transmission between populations, including recent spillover events in mammals (*14*,*15*).

The current understanding of HPAI virus transmission in wild birds involves circulation in migratory waterfowl or roving gulls (*6*) and mass mortality events within seabird colonies (*3*,*5*). Our study suggests that limited outbreaks in seabird colonies could play an important role in HPAI transmission chains. Post hoc surveillance of mass mortality is insufficient if seabird colonies can circulate HPAI without mass mortality. Therefore, we propose that more proactive monitoring of seabirds for HPAI virus infections will be critical for guarding commercial poultry (*1*), averting potentially catastrophic zoonotic transmission (*2*), and protecting vulnerable seabirds, including gulls.

Appendix Additional information for limited outbreak of highly pathogenic influenza A(H5N1) in herring gull colony, Canada, 2022
