# Supplementary material for: Limited Outbreak of Highly Pathogenic Influenza A(H5N1) in Herring Gull Colony, Canada, 2022
Source: Emerg Infect Dis. 2023 Oct;29(10):2150–4. doi: 10.3201/eid2910.230536 (PMC10521612; doi:10.3201/eid2910.230536)
Supplement: Appendix — Additional information for limited outbreak of highly pathogenic influenza A(H5N1) in herring gull colony, Canada, 2022 [file 23-0536-Techapp-s1.pdf]

Article DOI: https://doi.org/10.3201/eid2910.230536
EID cannot ensure accessibility for supplementary materials supplied by authors. Readers who have difficulty accessing supplementary content should contact the authors for assistance.

Limited Outbreak of Highly Pathogenic Influenza A(H5N1) in Herring Gull Colony, Canada, 2022

Appendix

Additional Methods

Case details are shown for the highly pathogenic avian influenza (HPAI) outbreak in American herring gulls (*Larus argentatus* subsp. *smithsonianus*) on Kent Island, New Brunswick, Canada, summer 2022 (Appendix Table 1). Images and videos of carcasses and live, symptomatic cases are available from a Dryad open-access repository (https://doi.org/10.5061/dryad.z08kprrjz).

Laboratory Testing

Six herring gull carcasses from Kent Island were collected for laboratory genetic testing for avian influenza virus, comprising 3 adults (cases 8, 20, and an additional euthanized adult) and 3 chicks (cases C1–C3). Initial sampling was performed by the Canadian Wildlife Health Cooperative, Atlantic Region. For each carcass, oropharyngeal and cloacal swab samples were collected and combined in viral transport media (Multitrans Transport System; Starplex Scientific, https://starplexscientific.com). PCR testing on those samples was performed at the Regional Diagnostic Virology Laboratory, Atlantic Veterinary College, University of Prince Edward Island, Canada. Samples were tested according to the Canadian Animal Health Surveillance Network protocol (Detection of Type A Influenza Viruses and Avian H5 and H7 Hemagglutinin Subtypes by Real-Time Reverse Transcription PCR [RT-PCR]). This protocol involves an initial avian influenza A matrix gene RT-PCR, then strain-specific RT-PCR to determine H5 or H7 subtypes.

Samples from 4 carcasses were positive for influenza A matrix and H5 subtype (adult cases 8, 20, and the euthanized bird along with chick case C2). All samples were negative for H7 influenza virus. Influenza A virus was not detected in chick cases C1 and C3. Positive samples (cases 8, 20, C2, and the euthanized bird) were sent to the National Centre for Foreign Animal Diseases, Canadian Food Inspection Agency (CFIA) for confirmatory testing (PCR, virus isolation, and genetic sequencing); testing identified a fully Eurasian lineage of influenza A(H5N1) in all 4 cases.

CFIA test results are accessible from the general public dashboard (https://cfia-ncr.maps.arcgis.com/apps/dashboards/89c779e98cdf492c899df23e1c38fdbc). To navigate to the case results for this study, in the dashboard address bar, search for: Kent Island, NB, CAN. Test results for herring gulls from Kent Island were listed by CFIA under collection dates that were different from true field collection dates and are not provided in a recognizable order (Appendix Table 2); however, those 4 cases correspond to the only 4 herring gull specimens submitted to CFIA from Kent Island (our 4 positive cases: 8, 20, C2, and the euthanized bird).

Adult Herring Gull Case Details

Case 1. Definitive-plumaged (≥4 years old) American herring gull (*L. a. smithsonianus*); East Beach, Kent Island, New Brunswick, Canada [latitude 44.578253, longitude −66.755109]; (2022–06–27, ≈09:30) first seen lethargic with drooped wings; (2022–06–27, ≈16:30) found dead following heavy rain, carcass with abdominal wound; carcass in mid-beach region, above tideline and below nesting area; (2022–07–04, 09:22; photo) noted undisturbed carcass; (2022–07–05, ≈09:00) noted undisturbed carcass.

Case 2. Definitive-plumaged (≥4 years old) American herring gull (*L. a. smithsonianus*); East Beach, Kent Island, New Brunswick, Canada [44.578507, −66.755071]; (2022–06–28, ≈17:00) first seen dead, carcass bent and spot of blood on stomach feathers but otherwise intact; carcass in mid-beach region, above tideline and below nesting area; 1 wing collected 36 hours after death; (2022–07–04, 09:18; photo) noted undisturbed carcass with abdominal wound, decomposing; (2022–07–06, 11:49; photo) noted undisturbed carcass.

Case 3. Number was used for a great black-backed gull.

Case 4. Definitive plumage with minor predefinitive plumage elements (≈4 years old), American herring gull (*L. a. smithsonianus*); West Beach, Kent Island, New Brunswick, Canada [44.585663, −66.763724]; (2022–07–01, 11:45) first seen dead, carcass huddled stomach down; carcass in low beach region, below tideline; (2022–07–04, 10:20) carcass missing (washed away?).

Case 5. Definitive-plumaged (≥4 years old) American herring gull (*L. a. smithsonianus*); West Beach, Kent Island, New Brunswick, Canada [44.583981, −66.762365]; (2022–07–01, 16:18) first seen lethargic with drooped wings; (2022–07–01, 17:20) found dead slightly higher up on beach from original position, stomach down; carcass in mid-beach region, above tideline and below nesting area; (2022–07–04, 10:15; photo) noted undisturbed carcass; (2022–07–05, 9:35; photo) noted undisturbed carcass; (2022–07–06, 12:34; photo) noted undisturbed carcass. Field notes record how carcass was ignored by nearby herring gulls, 2 of which walked near carcass (<2 m) when flushed during a census.

Case 6. Definitive-plumaged (≥4 years old) American herring gull (*L. a. smithsonianus*); East Beach, Kent Island, New Brunswick, Canada [44.5809163, −66.7542265]; (2022–07–02, 12:11) first seen lethargic with drooped wings; (2022–07–03 09:38) found dead near original location; carcass in mid-beach region, above tideline and below nesting area; (2022–07–04, 09:06; photo) noted undisturbed carcass; (2022–07–05, ≈09:00) noted undisturbed carcass; (2022–07–06, 11:31; photo) noted undisturbed carcass.

Case 7. Offsite. Definitive-plumaged (≥4 years old) American herring gull (*L. a. smithsonianus*); northwest Kent Island, New Brunswick, Canada [44.584545, −66.754029]; (2022–07–02, 14:48) first seen dead.

Case 8. Confirmed avian influenza virus. Definitive-plumaged (≥4 years old) American herring gull (*L. a. smithsonianus*); East Beach, Kent Island, New Brunswick, Canada [44.575913, −66.75488]; (2022–07–03, 09:49; video) first seen stationary and unresponsive with continuous head ticking; (2022–07–03, 16:42) alive in same spot, head ticking continues. Field notes record another slightly smaller herring gull in good health performing courtship or pair-bond behaviors and standing within inches of focal bird, alarmed at observer but back turned comfortably toward focal bird; head-nod toward focal bird (i.e., possible courtship display or food-begging); (2022–07–04, 09:30; video) alive in same spot, with head sagging down but still

softly ticking in identical repetitive movement; (2022–07–04, 14:18; photo) alive in same spot, eyes nearly closed, body hunched, and head barely but continuously ticking; (2022–07–05, 09:01) alive in same spot, fully collapsed with bill stuck in sand, immobile but breathing evident; (2022–07–05, 13:22) alive near same spot, now on back with eyes wide and tracking movement, feet kicking up in air; (2022–07–05, 17:52; video) found dead and carcass collected immediately for laboratory testing; carcass in mid-beach region, above tideline and below nesting area. Laboratory test results were confirmed positive for fully Eurasian avian influenza A(H5N1) virus.

Case 9. Definitive-plumaged (≥4 years old) American herring gull (*L. a. smithsonianus*); West Beach, Kent Island, New Brunswick, Canada [44.584552, −66.76353]; (2022–07–03, 16:41) first seen with severe loss of coordination, stumbling and flying toward water, then collapsing among intertidal boulders; attacked by nearby gulls while flailing in intertidal boulders; (2022–07–04, 10:20) found dead in same spot among rocks; carcass below the tideline.

Case 10. Definitive-plumaged (≥4 years old) American herring gull (*L. a. smithsonianus*); West Beach, Kent Island, New Brunswick, Canada [44.585997, −66.763762]; (2022–07–03, 16:43) same as adjacent case 9, first seen with severe loss of coordination, stumbling and flying toward water; attacked by nearby gulls while flailing and attempting to land in intertidal area; (2022–07–04, 10:25; photo) found dead, washed above tideline near previous flailing location; carcass chest cavity exposed (chest cavity eaten or rapidly decomposed during time in water?); (2022–07–05, 09:51; photo) noted undisturbed carcass; (2022–07–06, 12:36; photo) noted undisturbed carcass.

Case 11. Definitive-plumaged (≥4 years old) American herring gull (*L. a. smithsonianus*); East Beach, Kent Island, New Brunswick, Canada [44.581168, −66.754012]; (2022–07–03, 20:30) first seen collapsed, wings partially spread; (2022–07–04 08:57) found dead in same spot, carcass undisturbed; carcass in mid-beach region, above tideline and below nesting area; (2022–07–04, 13:28; photo) noted undisturbed carcass; (2022–07–06, 11:30; photo) noted undisturbed carcass.

Case 12. Because of tracking difficulties, possible interchanges with case 18. Definitive-plumaged (≥4 years old) American herring gull (*L. a. smithsonianus*); East Beach, Kent Island, New Brunswick, Canada [44.5788778, −66.7548414]; (2022–07–04, 09:13; video) first seen

lethargic with drooped wings, responsive to disturbance and nearby birds but slow and stumbling movement; (2022–07–04, 14:10) alive nearby, having moved from the mid-beach area to the tideline, head active but body wobbling and stiff; (2022–07–05, 08:46; photo) found dead near previous location, carcass just below tideline; (2022–07–06, 11:41; photo) noted undisturbed carcass.

Case 13. Definitive-plumaged (≥4 years old) American herring gull (*L. a. smithsonianus*); West Beach, Kent Island, New Brunswick, Canada [44.583007, −66.760653]; (2022–07–04, 10:04; video) first seen immobile, face down in sand with wings partially spread, open and responsive eyes; (2022–07–04, 14:49; photo) found dead in same spot, carcass near nesting area; (2022–07–06, 12:32; photo) noted undisturbed carcass.

Case 14. Definitive-plumaged (≥4 years old) American herring gull (*L. a. smithsonianus*); West Beach, Kent Island, New Brunswick, Canada [44.583888, −66.762139]; (2022–07–04, 10:11; photo) first seen dead with fully intact carcass, carcass near nesting area; (2022–07–04, 14:54) noted undisturbed carcass with blue-black flies swarming eyes; (2022–07–05, 09:35; photo) noted undisturbed carcass. (2022–07–06, 12:34; photo) noted undisturbed carcass.

Case 15. Definitive-plumaged (≥4 years old) American herring gull (*L. a. smithsonianus*); East Beach, Kent Island, New Brunswick, Canada [≈44.579046, −66.754931]; (2022–07–04, 13:51) first seen floating in water with spread wings, ruffled body but active head gazing toward a nearby, unresponsive flock of female common eiders (*Somateria mollissima*); (2022–07–04, 17:09) putative same individual seen alive at tideline, straight in from original floating location, lethargic and rocking back-and-forth with partially spread wings and narrow eyes; missing afterwards.

Case 16. Definitive-plumaged (≥4 years old) American herring gull (*L. a. smithsonianus*); East Beach, Kent Island, New Brunswick, Canada [44.575815, −66.75478982]; (2022–07–04, 14:21) first seen lethargic, head alert but no body coordination while stuck among rocks at edge of intertidal area; (2022–07–04, 17:23) seen alive but even less steady with bent neck among intertidal rocks; (2022–07–05, 09:05) seen alive in same spot; (2022–07–05, 17:54; video) seen alive, lethargic and wet with wings held close to body; (2022–07–06, 12:01) found dead near original location, carcass below the tideline.

Case 17. Offsite. Definitive-plumaged (≥4 years old) American herring gull (*L. a. smithsonianus*); South Basin, Kent Island, New Brunswick, Canada [44.5897, −66.75967]; (2022–07–04, 15:14; video) first seen flailing wings among group of ≈8 herring gulls. Flailing induced the other birds to panic and fly circles around flailing bird that was not coordinated enough to raise wings. As observer approached, focal bird found a way to take off, half flying and half carried by strong wings until it regained some composure higher in the air. Not seen again (impossible to confirm individual).

Case 18. Because of tracking difficulties, possible interchanges with case 12. Definitive plumage with minor predefinitive plumage elements (≈4 years old), American herring gull (*L. a. smithsonianus*); East Beach, Kent Island, New Brunswick, Canada [44.578673, −66.755152]; (2022–07–04, 17:13; photo) first seen dead with wings folded and head down, carcass near nesting area; (2022–07–05, ≈09:00) noted undisturbed carcass; (2022–07–06, 11:42; photo) noted undisturbed carcass.

Case 19. Definitive-plumaged (≥4 years old) American herring gull (*L. a. smithsonianus*); West Beach, Kent Island, New Brunswick, Canada [44.584494, −66.763426]; (2022–07–05, 09:46; photo) first seen dead with upper body intact but lower body ripped apart, carcass below tideline.

CASE 20. Confirmed avian influenza virus. Definitive-plumaged (≥4 years old) American herring gull (*L. a. smithsonianus*); East Beach, Kent Island, New Brunswick, Canada [44.578598, −66.754922]; (2022–07–05, 08:51) first seen with mobile head but uncoordinated body, stumbling, and unable to raise wings. Flailing from focal bird caused several dozen nearby herring gulls to flush with wild alarm, circling and screaming within 20 m; (2022–07–05, 13:15) seen alive near dense nesting area, wings spread and barely mobile; (2022–07–06, 11:37; video) seen alive having moved partially closer to tideline, eyes open and responsive but otherwise completely immobile; (2022–07–06, 16:46) seen alive in the same location, immobile with eyes wide open and still tracking movement; (2022–07–06, 19:39; photo) found dead and carcass collected immediately for pathology; carcass in mid-beach region, above tideline and below nesting area. Laboratory test results were confirmed positive for fully Eurasian avian influenza A(H5N1) virus.

Case 21. Number was skipped during field organization.

Case 22. Definitive-plumaged (≥4 years old) American herring gull (*L. a. smithsonianus*); West Beach, Kent Island, New Brunswick, Canada [44.583586, −66.7617]; (2022–07–05, 17:15; photo) found dead, soaking wet with stained but otherwise undisturbed carcass. Carcass was at tideline.

Case 23. Number was skipped during field organization.

Case 24. Definitive-plumaged (≥4 years old) American herring gull (*L. a. smithsonianus*); East Beach, Kent Island, New Brunswick, Canada [44.580714, −66.754379]; (2022–07–05, 17:45; video) first seen flailing in high-tide water causing alarm among nearby gulls; (2022–07–05, 18:18; video) after crawling on shore, flipped sideways with wing stuck in the air, quivering legs and active eye; (2022–07–06, 11:34; photo) found dead in same spot, carcass below tideline.

Case 25. Definitive-plumaged (≥4 years old) American herring gull (*L. a. smithsonianus*); West Beach, Kent Island, New Brunswick, Canada [44.584865, −66.764062]; (2022–07–05, 16:20) found dead, carcass floating in water off beach. Confirmation of other monitored carcasses nearby supports new case.

Case 26. Missing. Definitive-plumaged (≥4 years old) American herring gull (*L. a. smithsonianus*); East Beach, Kent Island, New Brunswick, Canada [44.580752, −66.75464]; (2022–07–05, 17:10) first seen lethargic and wet high up in berm among dense nesting area; (2022–07–06, 11:35; video) seen again in same territory, lethargic, slumped in shrub, eyes nearly closed, wet head and body after rains; (2022–07–06, 16:49) noted absent with 3 dead chicks found within 4 m of each other on the same territory where focal bird was previously loafing (1 chick collected [case C2], which tested positive for HPAI A virus).

Case 27. Recovered? Definitive-plumage with minor predefinitive plumage elements (≈4 years old), American herring gull (*L. a. smithsonianus*); East Beach, Kent Island, New Brunswick, Canada [44.5776, −66.75518]; (2022–07–06, 11:54; video) first seen fully flailing and unable to lift wings, prompting many nearby gulls to rise in alarm; (2022–07–6, 16:42; video) seen ≈20 m from original spot but now coordinated enough to hold wings and walk; (2022–07–06, 19:53) seen in same spot as previous observation, now fully mobile and, after a few steps, able to fly down the beach; subsequently lost from monitoring. Although it is not

obvious in all recorded media, we were able to identify this bird more confidently across sightings via a distinct predefinitive plumage element (black primary coverts).

Case 28. Definitive-plumage with minor predefinitive plumage elements (≈4 years old), American herring gull (*L. a. smithsonianus*); East Beach, Kent Island, New Brunswick, Canada [44.576526, −66.75514]; (2022–07–06, 11:58; photo) first seen dead face down in sand and undisturbed; carcass in mid-beach region, above tideline and below nesting area.

Case 29. Offsite. Definitive-plumaged (≥4 years old) American herring gull (*L. a. smithsonianus*); South Basin, Kent Island, New Brunswick, Canada [44.58616, −66.76336]; (2022–07–07, 11:03; photo) first seen dead, carcass clearly disturbed with chest wound and scattered feathers among intertidal rocks.

Case 30. Offsite. Definitive-plumaged (≥4 years old) American herring gull (*L. a. smithsonianus*); South Basin, Kent Island, New Brunswick, Canada [44.587328, −66.759744]; (2022–07–7, 11:10; photo) first seen dead, carcass wet but undisturbed among intertidal rocks.

Case 31. Definitive-plumaged (≥4 years old) American herring gull (*L. a. smithsonianus*); East Beach, Kent Island, New Brunswick, Canada [44.580715, −66.754472]; (2022–07–08, 16:23; video) first seen lethargic and hunched in intertidal area; (2022–07–08, 19:50; photo) found dead near original location, carcass below tideline; (2022–07–09, 16:41; photo) noted undisturbed carcass.

Case 32. Definitive-plumaged (≥4 years old) American herring gull (*L. a. smithsonianus*); East Beach, Kent Island, New Brunswick, Canada [44.581122, −66.753938]; (2022–07–10, 16:30; video) first seen uncoordinated and trembling close to water; (2022–07–10, 19:45; video) seen again immobile, floating at edge of water; (2022–07–11, 16:42; photo) found dead, carcass below tideline.

Case 33. Definitive-plumaged (≥4 years old) American herring gull (*L. a. smithsonianus*); East Beach, Kent Island, New Brunswick, Canada [44.581909, −66.7538]; (2022–07–15, 16:06; photo) found dead; carcass in mid-beach region above tideline and below nesting area.

Case 34. Definitive-plumaged (≥4 years old) American herring gull (*L. a. smithsonianus*); East Beach, Kent Island, New Brunswick, Canada [44.581141, −66.754134]; (2022–07–17, 16:25; photo) first seen dead having washed up on beach, carcass at tideline.

Case 35. Definitive-plumaged (≥4 years old) American herring gull (*L. a. smithsonianus*); East Beach, Kent Island, New Brunswick, Canada [44.583043, −66.753943]; (2022–07–19, 16:30; photo) found dead, carcass near nesting area.

Case 36. Definitive-plumaged (≥4 years old) American herring gull (*L. a. smithsonianus*); East Beach, Kent Island, New Brunswick, Canada [44.583052, −66.753972]; (2022–07–21, 7:39; video) first seen lethargic and uncoordinated in nesting area; (2022–07–21, 16:26; video) seen alive in same spot, head mobile but otherwise collapsed; (2022–07–22, 9:46) found dead in same location among nesting territories; (2022–07–26, 10:22; photo) noted undisturbed carcass.

Case 37. Definitive-plumaged (≥4 years old) American herring gull (*L. a. smithsonianus*); East Beach, Kent Island, New Brunswick, Canada [44.580984, −66.75419]; (2022–07–25, 13:45) first seen minorly lethargic, wings drooped and ruffled feathers but mobile and coordinated wing movements; (2022–07–26, 10:28; photo) found dead, carcass below tideline; (2022–07–27, 10:09) carcass noted missing, possibly swept away by tide.

Case 38. Definitive-plumaged (≥4 years old) American herring gull (*L. a. smithsonianus*); East Beach, Kent Island, New Brunswick, Canada [44.580984, −66.75419]; (2022–07–26, 10:25; video) first seen with uncoordinated movement. Not seen for multiple days; (2022–07–29, 8:56) found dead after thorough search a few meters from original live location, carcass intact but drenched from heavy rains. Carcass was in nesting area.

Case 39. First-cycle plumage (≈1 year old), America herring gull (*L. a. smithsonianus*); East Beach, Kent Island, New Brunswick, Canada [44.581149, −66.75398]; (2022–08–08, 10:13; photo) first seen dead face down in sand, carcass wet but otherwise intact. Carcass was at tideline.

Case 40. First-cycle plumage (≈1 year old), America herring gull (*L. a. smithsonianus*); East Beach, Kent Island, New Brunswick, Canada [44.582075, −66.753745]; (2022–08–12,

8:13) first seen dead with heavily decomposed carcass suggesting earlier death. Carcass was at tideline.

Chick Herring Gull Case Details

Case C1. Negative for HPAI A virus. Recently hatched (≈1 month old) American herring gull chick (*L. a. smithsonianus*); in field nesting territory on South Field, Kent Island, New Brunswick, Canada [44.581384, −66.755459]; some difficulties tracking individual chick across multiple sightings; first accurately noted (2022–07–06, ≈13:30), seen dry and upright, but slow moving and not fleeing from observer, 1 definitive-plumaged parent was closely attending; (2022–07–06, 16:07) seen dry and upright but more stationary; (2022–07–06, 17:28; video) dry and walking with stumbling and uncoordinated movements; (2022–07–07, 12:16) found dead with healthy parent in close attendance (<2 m from chick, alarmed at and defensive with observer). Carcass was collected for testing. Laboratory testing was negative for HPAI virus.

Case C2. Confirmed HPAI A virus. Recently hatched (≈1 month old) American herring gull chick (*L. a. smithsonianus*), putative offspring of case 26, found in nesting territory where that adult bird was seen ill; East Beach, Kent Island, New Brunswick, Canada [44.580752, −66.75464]; (2022–07–06, 16:49; photo) first seen dead with 2 other chicks (<4 m apart), all carcasses unattended but fully intact. Carcass was collected for testing; laboratory testing was confirmed positive for fully Eurasian avian influenza A(H5N1) virus.

Case C3. Negative for HPAI A virus. Recently hatched (≈1 month old) American herring gull chick (*L. a. smithsonianus*); in field nesting territory on South Field, Kent Island, New Brunswick, Canada [44.580765, −66.755776]; (2022–07–07, 12:52) first seen dead, carcass fully intact and parent closely attending. Carcass was collected immediately for testing; laboratory testing returned negative for HPAI virus.

Putative HPAI in Other Kent Island Species

Great Black-Backed Gull (*Larus marinus*)

One closely-monitored putative HPAI case in great black-backed gull (*L. marinus*); definitive-plumaged adult (≥4 years old) on East Beach, Kent Island, New Brunswick, Canada [44.575719, −66.754705]; (2022–06–29, 16:20; photo) first seen severely uncoordinated in intertidal area, where flailing caused mob of other gulls to rise up nearby; observed multiple

times over subsequent hours, recording how flailing diminished to complete immobility despite open and active eyes; (2022–06–30, 9:22) found dead with wet carcass (drowned overnight?); (2022–07–04, 9:37; photo) noted undisturbed carcass; (2022–07–04, 17:20; photo) noted carcass disturbance with body in new position, feathers strewn about, and body cavity peeled open. (2022–07–05, ≈9:00) noted undisturbed carcass. Additional *L. marinus* deaths found across Kent Island (2 carcasses, definitive plumage, ≥4 years old), Sheep Island (7 carcasses, definitive plumage, ≥4 years old), and Hay Island (1 carcass, first-cycle plumage, ≈1 year old).

Canada Goose (*Branta canadensis*)

At least 3 adult *B. canadensis* carcasses found across Kent Island. Carcasses were generally decomposed but otherwise intact.

Common Eider (*Somateria mollissima*)

One adult female *S. mollissima* was reported lethargic and curled up on West Beach, Kent Island, later found dead with intact carcass. Another intact female carcass was found on Hay Island. Regional monitoring confirmed fully Eurasian avian influenza A(H5N1) virus in 2 carcasses from nearby Grand Manan, New Brunswick, Canada (1 collected on 2022–06–18; 1 collected on 2022–07–03) (https://cfia-ncr.maps.arcgis.com/apps/dashboards/89c779e98cdf492c899df23e1c38fdbc).

American Crow (*Corvus brachyrhynchos*)

A living adult crow was seen uncoordinated and flailing in intertidal area just south of East Beach, Kent Island. As with flailing herring gulls, the flailing crow drew aggressive mobbing behaviors from nearby gulls (video). At least 3 additional intact carcasses were reported across Kent Island (photo). Laboratory testing confirmed fully Eurasian avian influenza A(H5N1) virus infection in 1 American crow carcass from Kent Island (2022–07–10; https://cfia-ncr.maps.arcgis.com/apps/dashboards/89c779e98cdf492c899df23e1c38fdbc).

Other

HPAI cases from those above and other avian species (e.g., Arctic terns, *Sterna paradisaea*) were reported in the Bay of Fundy and Atlantic Canada (https://cfia-ncr.maps.arcgis.com/apps/dashboards/89c779e98cdf492c899df23e1c38fdbc). Although black

guillemots (*Cepphus grylle*) and Leach's storm petrels (*Hydrobates leucorhous*) also breed among herring gulls on Kent Island, we saw no evidence of HPAI in those species.

Appendix Table 1. Summary of herring gull locations and carcass status for putative highly pathogenic avian influenza cases on the East and West beach study areas of American herring gull colony on Kent Island, New Brunswick, Canada*

Case	Beach	Latitude	Longitude	Area†	Media	Carcass status	Carcass disturbance‡
1	East	44.578253	−66.755109	Mid	Dead	Abdominal wound	Undisturbed ≥7.7 d
2	East	44.578507	−66.755071	Mid	Dead	Abdominal wound	Undisturbed ≥7.9 d
6	East	44.5809163	−66.7542265	Mid	Dead	Intact	Undisturbed ≥3.1 d
8	East	44.575913	−66.75488	Mid	Live, Dead	Intact	Collected immediately
11	East	44.581168	−66.754012	Mid	Dead	Intact	Undisturbed ≥2.1 d
12	East	44.5788778	−66.7548414	Low	Live, Dead	Intact	Undisturbed ≥1.9 d
15	East	44.579046	−66.754931	Low	NA	Missing	NA
16	East	44.575815	−66.75478982	Low	Live, Dead	Intact	NA
18	East	44.578673	−66.755152	Nest	Dead	Intact	Undisturbed ≥1.8 d
20	East	44.578598	−66.754922	Mid	Live, Dead	Intact	Collected immediately
24	East	44.580714	−66.754379	Low	Live, Dead	Intact	NA
26	East	44.580752	−66.75464	Nest	Live	Missing	NA
27	East	44.5776	−66.75518	Mid	Live	*Recovered?*	NA
28	East	44.576526	−66.75514	Mid	Dead	Intact	NA
31	East	44.580715	−66.754472	Low	Live, Dead	Intact	Undisturbed ≥0.9 d
32	East	44.581122	−66.753938	Low	Live, Dead	Intact	NA
33	East	44.581909	−66.7538	Mid	Dead	Intact	NA
34	East	44.581141	−66.754134	Low	Dead	Intact	NA
35	East	44.583043	−66.753943	Nest	Dead	Intact	NA
36	East	44.583052	−66.753972	Nest	Live, Dead	Intact	Undisturbed ≥5.1 d
37	East	44.580984	−66.75419	Low	Dead	Intact	Carcass missing after 1.0 d
38	East	44.581273	−66.754193	Nest	Live	Intact	NA
39	East	44.581149	−66.75398	Low	Dead	Intact	NA
40	East	44.582075	−66.753745	Low	NA	Decomposed	NA
4	West	44.585663	−66.763724	Low	NA	Intact	NA
5	West	44.583981	−66.762365	Mid	Dead	Intact	Undisturbed ≥4.8 d
9	West	44.584552	−66.76353	Low	NA	Not recorded	
10	West	44.585997	−66.763762	Mid	Dead	Lower body missing	Undisturbed ≥2.1 d
13	West	44.583007	−66.760653	Nest	Live, Dead	Intact	Undisturbed ≥1.9 d
14	West	44.583888	−66.762139	Nest	Dead	Intact	Undisturbed ≥2.1 d
19	West	44.584494	−66.763426	Low	Dead	Lower body destroyed	NA
22	West	44.583586	−66.7617	Low	Dead	Intact	NA
25	West	44.584865	−66.764062	Low	NA	Intact	NA

*Locations approximate given GPS reception. NA, not applicable.

†Section of beach where the carcass or living bird was found. The nest area near the berm included all nests and breeding territories, the low area was at or below the tideline, and the mid area was in-between.

‡Carcass disturbance records simply indicate when monitoring ended; no herring gull carcass was clearly damaged after being discovered (but see case details for great black-backed gulls). Only 7/33 carcasses (27%) with beach location data were found in the nesting area farther from shore. The remaining birds either wandered or were forced toward the water (presumably by aggression from other birds) before dying. Some carcasses on Kent Island appeared with initial injuries but otherwise remained undisturbed (monitored 0.9–7.9 d). Carcasses monitored for disturbance remained untagged.

Appendix Table 2. Canadian Food Inspection Agency confirmatory testing for avian influenza A(H5N1) virus in 4 herring gull specimens submitted from Kent Island, New Brunswick, Canada, 2022

CFIA collection date	Result authorized	Common name	Province	Status	Strain	Result	Lineage
7/18/2022	8/10/2022	Herring gull	New Brunswick	Dead	H5N1	Confirmed	Fully Eurasian
7/5/2022	8/10/2022	Herring gull	New Brunswick	Dead	H5N1	Confirmed	Fully Eurasian
7/4/2022	8/10/2022	Herring gull	New Brunswick	Dead	H5N1	Confirmed	Fully Eurasian
7/2/2022	8/10/2022	Herring gull	New Brunswick	Dead	H5N1	Confirmed	Fully Eurasian
